# Activated omega loops for anterior crossbite correction in transitional dentition

**DOI:** 10.1002/ccr3.7685

**Published:** 2023-07-10

**Authors:** Adith Venugopal, Farooq Ahmed, Rajiv Yadav, Noem Bunthouen

**Affiliations:** ^1^ Department of Orthodontics University of Puthisastra Phnom Penh Cambodia; ^2^ Guy's & St Thomas' NHS Foundation Trust London England; ^3^ Department of Orthodontics and Dentofacial Orthopedics Tribhuvan university dental teaching hospital, Institute of medicine, Maharajgunj Medical Campus Kathmandu Nepal; ^4^ Private Practice Phnom Penh Cambodia

**Keywords:** lingual tipping, anterior crossbite, omega loops, pseudo‐class III

## Abstract

**Key Clinical Message:**

Several approaches can correct pseudo‐Class III anterior crossbite. 2 × 4 appliance, compressed open‐coil springs, Class III elastics, etc. All cause either soft tissue lacerations, smile line flattening, or upper incisor overproclination. This paper describes a novel method to tip lower incisors into a normal overjet without compromising the upper dentition.

**Abstract:**

In pseudo‐class III cases, a “two by four” multibracketed appliance has been utilized to put the incisors into a typical overjet during transitional dentition. Compressing a rectangular super elastic archwire creates continuous force, but its length restricts activation and risks cheek impingement. Open‐coil springs on rigid archwires advance incisors labially, although a 4‐5 mm of wire distal to the molar tube may injure soft tissue. Reciprocally anchored Class III intermaxillary elastics restore anterior overjet through lower incisor lingual tipping and upper incisor proclination. Class III elastics extrude maxillary molars and mandibular incisors, rotating the dental occlusal plane counterclockwise and reducing maxillary incisor exposure and aesthetics. A unique method is reported in this report to tip the lower incisors back into normal overjet without affecting the upper dentition.

## Graphical abstract

Activated omega loops for the lingual tipping of the mandibular incisors.

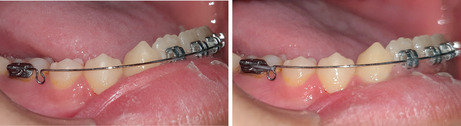

## 1 BACKGROUND

Anterior crossbite affects 2.2%–11.9% of pre‐adolescent patients.^1^, ^2^ During transitional dentition, a variety of orthodontic appliances and mechanics have been employed to move the incisors into a normal overjet. A multibracketed appliance, also known as a “two by four” appliance, is a standard approach‐involving incisor and molar brackets.^3^ Although the force generated by compressing a rectangular super elastic archwire is continuous, these mechanics offer limited activation through archwire length and the risk of cheek impingement. The additional stages of leveling and aligning are required prior to activation of the compressed archwire with the use of a two by four appliance. Alternatively, the incisors can be advanced labially by compressing open‐coil spring on a rigid archwire from incisor to molar tube. However, this method involves 4‐5 mm of wire extended distal of the molar tube, potentially injuring the soft tissue. Another option is the use of Class III intermaxillary elastics, utilizing reciprocal anchorage to correct the anterior relationships through lingual tipping of the lower incisors and proclination of the maxillary incisors. However, during treatment employing Class III elastics, the extrusion of maxillary molars and mandibular incisors rotates the dental occlusal plane counterclockwise, decreasing the maxillary incisor exposure and compromising the aesthetic outcomes.^4^, ^5^

## 2 CLINICAL TECHNIQUE

**FIGURE 1:**
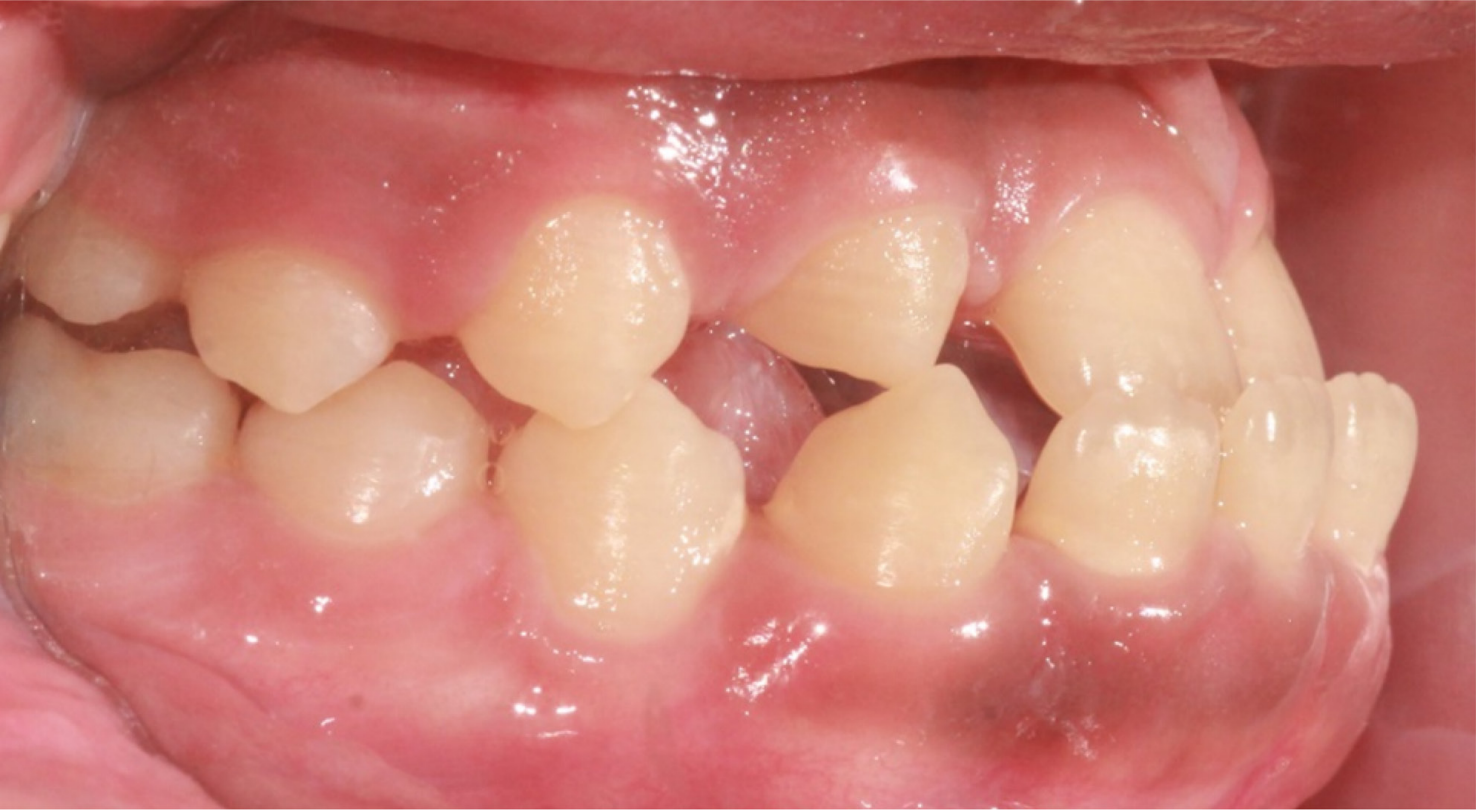
Transitional dentition with a pseudo‐class III malocclusion.

**FIGURE 2:**
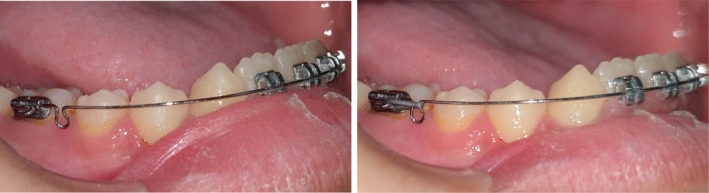
0.014”/0.016” SS archwire with an omega loop constructed about 2 mm mesial to the buccal tube in the passive form. Activation occurs through an elastic module tied back from the molar hook to the loop to activate the arch wire.

**FIGURE 3:**
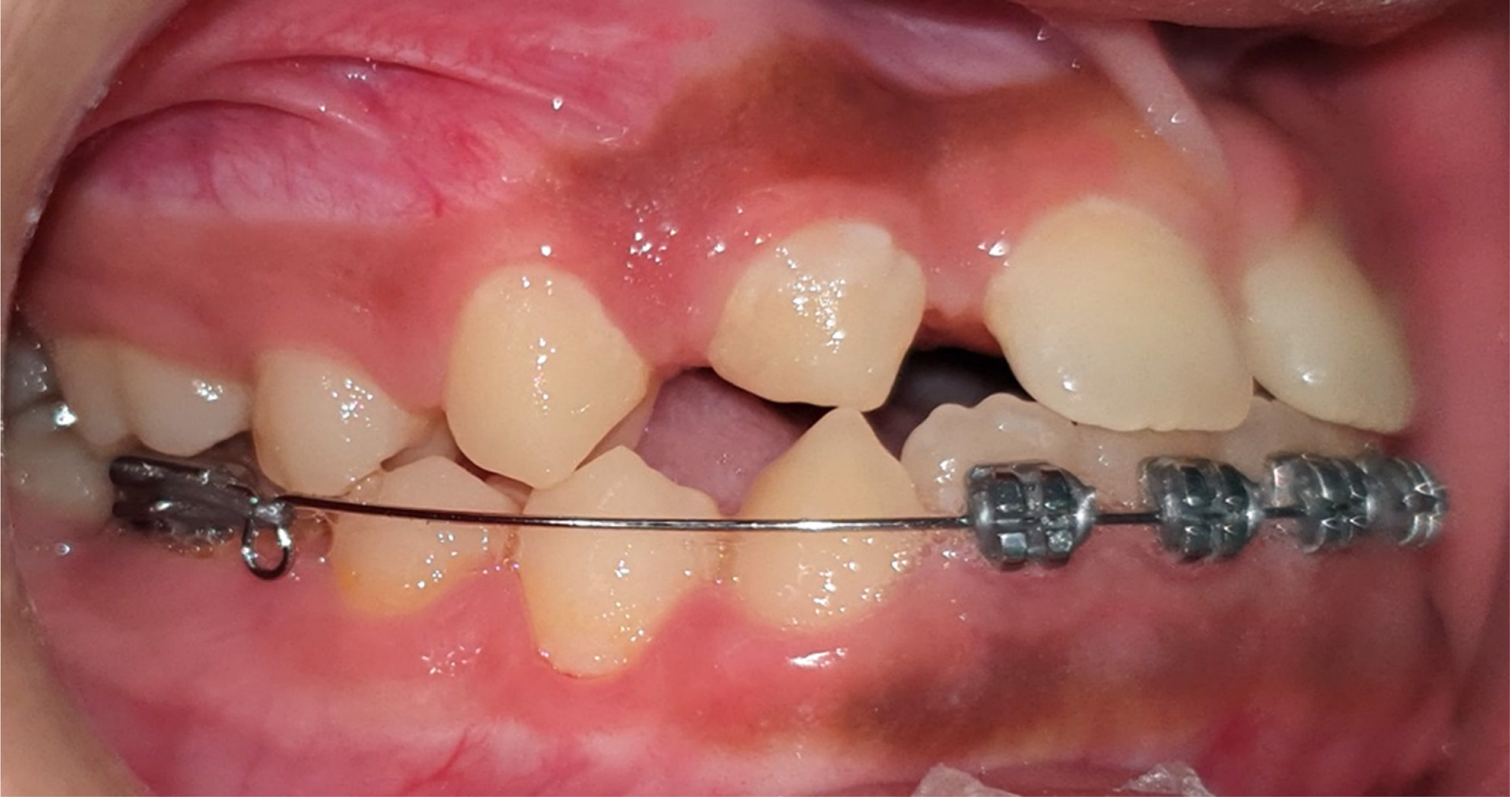
Lower anterior teeth tipped lingually and correction of the crossbite.

A 11‐year‐old boy with an anterior cross bite had spaces available distal to the lateral incisors (usually the case with transitional dentition). In Pseudo‐Class III malocclusions, the lower incisors are usually proclined (Figure 1). We used a 0.014/0.016” SS archwire with an omega loop constructed about two millimeters mesial to the buccal tube in the passive form (Figure 2). Activation occurs through an elastic module tied back from the molar hook to the loop to activate the arch wire (Figure 2), thereby tipping the anterior teeth lingually and correcting the crossbite (Figure 3).

**FIGURE 4:**
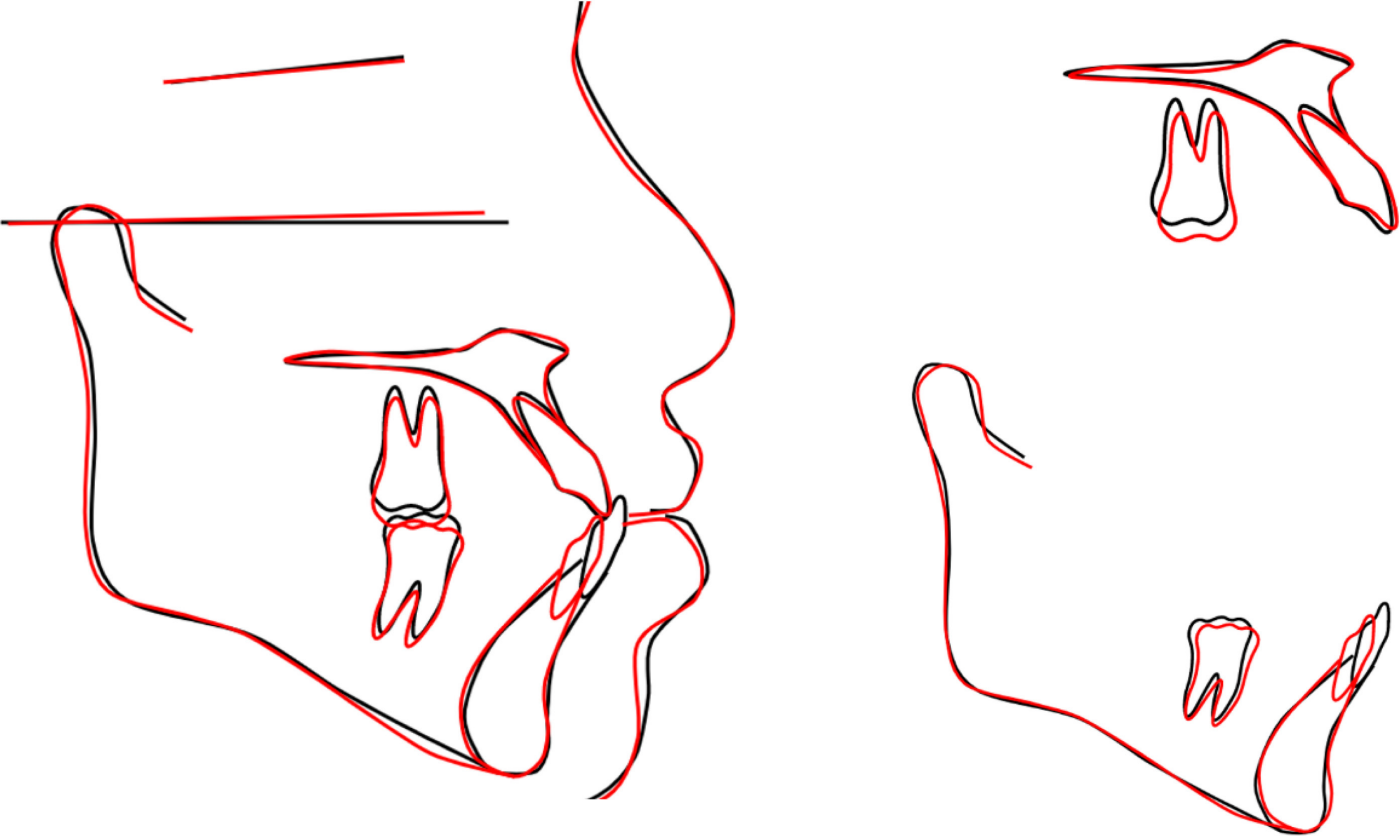
Cephalometric superimpositions.

**FIGURE 5:**
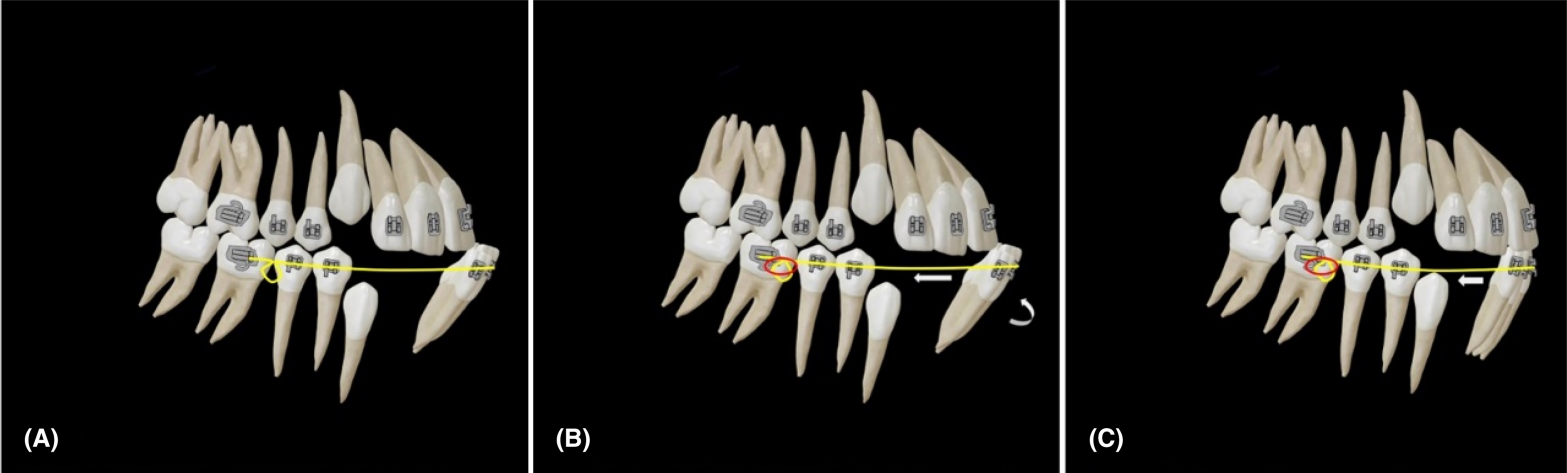
Graphical representation of the clinical technique describing the lingual tipping of lower incisors in transitional dentition cases.

The advantages include a reasonably simplistic and basic design with few side effects on the upper arch (such as excessive anterior proclination, which is usually performed to jump the bite). With such mechanics, slight mesial tipping of the lower molars and lingual tipping of the lower incisors can be anticipated (Figure 4). We were able to successfully correct the anterior crossbite in the patient presented using the aforementioned approach (Figure 5).

## AUTHOR CONTRIBUTIONS

**Adith Venugopal:** Conceptualization; investigation; writing – original draft. **Farooq Ahmed:** Writing – original draft; writing – review and editing. **Rajiv Yadav:** Writing – original draft; writing – review and editing. **Noem Bunthouen:** Conceptualization.

## FUNDING INFORMATION

No funding has been received to support this study.

## CONFLICT OF INTEREST STATEMENT

The authors declare no conflict of interest.

## CONSENT STATEMENT

Written informed consent was obtained from the patient to publish this report in accordance with the journal's patient consent policy.

## Data Availability

The data used to support the findings of this study are available from the corresponding author upon request.
